# Effect of Subsidies and Tax Deductions on Promoting the Construction of Long-Life Quality Houses in Japan

**DOI:** 10.3390/ijerph15112376

**Published:** 2018-10-26

**Authors:** Ken’ichi Matsumoto, Yuki Yamamoto, Nao Ohya

**Affiliations:** 1 Graduate School of Fisheries and Environmental Sciences, Nagasaki University, 1-14 Bunkyo-machi, Nagasaki 852-8521, Japan; y-yamamoto@nagasaki-u.ac.jp; 2 Faculty of Environmental Science, Nagasaki University, 1-14 Bunkyo-machi, Nagasaki 852-8521, Japan; jackie.0808n@gmail.com

**Keywords:** long-life quality housing, subsidies, tax deductions, panel data analysis, policy analysis, Japan

## Abstract

Securing a quantity of houses for citizens has been the priority of housing policies in Japan. However, these policies shifted from quantity to quality in the 21st century, including the promotion of “long-life quality housing (LLQH)”, which contributes to a sustainable and healthy society for the residential sector. Since then, various policies have been introduced at the national and prefectural (local) levels to promote the construction of LLQH. Using panel data for 47 prefectures across seven years, this study aims to analyze the factors that Japanese households choose when constructing LLQH. Although various research on LLQH and similar housing exists, this study is the first attempt to empirically explore the factors that promote LLQH. We found that policy measures covering only LLQH were generally effective in promoting the construction of LLQH, and these policy measures were more effective than those covering both LLQH and other types of housing. National-level measures tended to be effective, whereas prefectural-level measures were not. Furthermore, although the effects of individual measures differed, the overall effects of policy measures were confirmed. In conclusion, providing economic incentives had a positive effect on promoting LLQH, and such measures were successful in achieving the intended purpose.

## 1. Introduction

Housing policies in Japan after World War II have focused on the quantitative supply of houses to address the shortage of 4.2 million houses caused by damages from the war [1]. Moreover, during the country’s period of high economic growth from the late 1950s to the early 1970s, the housing shortage became a more serious issue because of population increases and the influx of people into cities. In response, the Japanese government hurried to increase the supply of housing. Before the war, it was a custom in Japan to build houses of good quality and use them for a long time, continually repairing any damage. However, during the period of high economic growth, securing large quantities of housing became the priority of the government. The lifetime of Japanese houses therefore became shorter than that in previous periods [1,2]. Compared with those of houses in the US (55 years) and the UK (77 years), the estimated average lifetime of Japanese houses is much shorter (30 years), although the nation’s natural and geographical conditions are different [3,4]. Furthermore, the share of existing housing distribution (including all types of housing, calculated by existing housing divided by the sum of existing housing and new housing in a year) in the Japanese housing market is 14.7% as of 2013, which is much lower than those in the US and in European countries (around 70–80%) [5].

This trend began to change in 2006, when the Basic Plan for Housing and the Basic Act for Housing were established. Based on the Basic Plan, the policy focus shifted to improving the quality of houses rather than ensuring a certain amount of housing, which represents a shift from a flow society to a stock society. Furthermore, the Act on the Promotion of Dissemination of Long-Life Quality Housing (the English translation of the Act differs across sources, but here, we used the translation in the White Paper on Land, Infrastructure, Transport and Tourism Japan 2016 [6]) was enacted in 2008 to promote the construction of high-quality houses with long lifetimes, as well as to eventually contribute to quality of life and sustainable development in the residential sector. Long-life quality housing (LLQH), a type of sustainable and healthy housing, consists of houses that meet standards, such as durability, ease of maintenance and management, absence of barriers, indoor air quality, and energy efficiency, enabling them to be used with consistent quality for a long time [5,6,7]; long-life housing is considered a sustainable building that uses fewer resources and can reduce CO_2_ emissions over the course of its life cycle [8]. The following criteria must be fulfilled to be certified as LLQH (newly built houses) [9] (the technical aspects of the criteria are available online [10]; some criteria are different for apartment-type housing).

- Measures against degradation: Level 3 (the highest criteria level in the Housing Performance Indication System) measures against degradation (Level 1: measures required by the Building Standards Act; Level 2: measures to extend the life of housing to 50–60 years [two generations]; and Level 3: measures to extend the life of housing to 75–90 years [three generations]).
- Seismic capacity: Level 1 or 2 seismic capacity or a seismically isolated structure (Level 1: seismic capacity required by the Building Standards Act; Level 2: seismic capacity 1.25 times higher than that required by the Building Standards Act; and Level 3: seismic capacity 1.5 times higher than that required by the Building Standards Act).
- Ease of management and renewal: level 3 (the highest criteria level in the Housing Performance Indication System) operation and maintenance measures (Level 1: other than levels 2 and 3; Level 2: basic measures for easy management and renewal [e.g., not embedding pipes into concrete]; and Level 3: specific measures for easy management and renewal [e.g., installing cleaning holes and inspection chambers]).
- Energy saving: Level 4 (the highest criteria level in the Housing Performance Indication System) heat insulation capacity (Level 1: other than levels 2–4; Level 2: measures to save a small amount of energy [energy saving standard established in 1980]; Level 3: measures to save a moderate amount of energy [energy saving standard established in 1992]; and Level 4: measures to save a large amount of energy, as required by the Act on the Rational Use of Energy [energy saving standard established in 2016]).
- Living space: 75 m^2^ or larger.
- Living environment: Harmonization with district planning, landscape planning, building agreement, etc.
- Plan for maintenance: Development of a plan for future periodic inspections and maintenance of housing.

The main expected advantages of building LLQH are threefold: (1) suppression of waste discharge from housing demolitions, (2) reduction of environmental burdens, and (3) mitigation of the financial burden of residents through a reduction in costs associated with building reconstruction [7]. Because of these aspects, constructing more LLQH will help Japan in moving toward a more sustainable society, in terms of environmental and living conditions, for the residential sector. LLQH could also contribute to solving environmental issues and health-related problems from the energy perspective. This is attributed to the characteristics of LLQH, specifically its high heat insulation performance [3,9]. However, because of these characteristics, the construction costs of LLQH are higher than those of regular housing [3]. This means that monetary incentives and similar policies are required to stimulate the LLQH market in Japan.

Studies related to long-life housing and associated topics in Japan examine the lifetimes of houses [11], their environmental burdens [12], policies related to the extension of their lifetimes and energy-saving abilities [13], the energy and environmental impacts of high-performance solar houses [14], the construction and evaluation of low-energy housing [15], and the energy management of zero-energy house communities [16]. However, the number of studies related to LLQH is limited, including only a qualitative analysis of an initiative LLQH model project [17], a case study on the conditions and rules for securing housing environments for LLQH [18], and studies on stakeholders’ perspectives of a building environmental assessment method [19].

There are also studies on similar types of housing in other countries. For example, Park and Tae [20] compared the housing policies of 10 countries on three aspects related to the quality of housing and offered policy suggestions to improve the quality of housing in Korea. Other studies include an evaluation of the scores and status of housing under the certification system for long-life housing in Korea [21], an evaluation of the lifecycle CO_2_ emission reduction of long-life apartment housing in Korea [8], the development of regulations for Taiwan’s sustainable building (open building) [22], and a survey of stakeholders’ preferences for the “soft” features of sustainable and healthy housing in the UK [23]. In addition, a similar housing concept (e.g., sustainable housing) has also been studied from various perspectives, but the focus has been on design [24,25], affordability [26,27], community [28,29], urban construction [30,31], climate change mitigation [32], and energy savings and consumption [33,34]. There are also studies on sustainable housing from the perspective of health (or health is considered a factor in the evaluations) [24,27,30,35,36].

While there are studies related to LLQH or similar types of housing, there are none that analyzed the factors and policy measures necessary to determine and increase the amount and share of such housing. Empirical studies with panel data have been conducted on households’ demand for and choice of housing [37,38,39,40,41], but these have focused on regular housing. Furthermore, although studies on the relationship between taxation (and taxation reform) and housing markets have been conducted [42,43,44], their targets have been regular housing. These studies were also performed more than 20 years ago. Elucidating the factors, particularly policy measures, contributing to the promotion of LLQH is important to efficiently facilitate the construction of LLQH. The purpose of this study, therefore, is to identify the factors needed to increase the share of LLQH, with a particular focus on the policy measures intended to support the construction of LLQH, using a panel data approach.

## 2. Materials and Methods

### 2.1. Policy Measures for Long-Life Quality Housing

Policy measures to promote the construction of LLQH have been introduced at both the national and prefectural (local) levels. 

#### 2.1.1. Policy Measures at the National Level

The Act on the Promotion of Dissemination of Long-life Quality Housing stipulates that national and local governments shall take fiscal, monetary, and other measures to promote LLQH. As a measure corresponding to this Act, a tax reform was implemented for LLQH in 2008 (Table 1; 1 JPY is equivalent to 0.0089 USD as of 21 September 2018). This was intended to reduce the burdens of households when building LLQH, the cost of which is expected to be 1.2 times higher than that for regular houses [45]. In addition, in the tax reform of 2009, the income tax deduction for households with housing loans for LLQH was largely increased (Table 2). A policy was also introduced in 2009 to deduct income tax equal to 10% of the amount of the enhancement of house performance (maximum of one million JPY in 2009–2011, 500 thousand JPY in 2012–2014, and 650 thousand JPY since 2015; these years show when residents started living in the houses) when a household builds or purchases LLQH without taking a housing loan. 

In addition to these preferential measures, Japan’s national government has provided subsidies for building LLQH. These subsidies started in 2009 and have continued up to the present time, although the schemes have been updated intermittently [46,47,48,49]. The subsidies amounted to one million JPY from 2009 to 2014 and increased to 1.3 million JPY in 2015 and 1.5 million JPY since 2016.

In addition to these subsidies that only cover LLQH, other schemes exist to support building both LLQH and houses, such as highly insulated houses (these houses meet a part of the conditions for LLQH). These include Subsidies for Housing Stock Circulation Support, Housing Cash Benefit, and Housing Eco-Points [50,51,52]. Subsidies for Housing Stock Circulation Support, which started in 2016, provides subsidies of 500 thousand JPY. Housing Cash Benefit started in 2014 to reduce the burden of increases in consumption taxes for those building houses, including LLQH, and provides subsidies of 300 thousand JPY. Housing Eco-Points, implemented from 2010 to 2014, awarded points to houses that meet energy-saving standards; these points could be exchanged for environment-friendly and economy-revitalizing products, such as high-efficiency home electrical appliances. 

#### 2.1.2. Policy Measures at the Prefectural Level

Policy measures covering only LLQH are limited at the prefectural level. Since 2012, Fukuoka Prefecture has had its own scheme, the Project to Promote Long-Life Quality Houses in Fukuoka, to support LLQH [53]. In this policy measure, the Prefecture cooperates with private financial institutions to reduce the housing loan rates or loan fees for households building LLQH. A similar scheme has also been implemented in Shizuoka Prefecture since 2009 [54].

Policy measures at the prefectural level that cover both LLQH and other types of housing (e.g., highly insulated houses) are broader but still limited. These schemes have been introduced in the following six prefectures: Iwate (from 2010) [55], Ishikawa (from 2012) [56], Nagano (from 2010) [57], Gifu (from 2011) [58], Kochi (from 2015) [59], and Fukuoka (from 2009) [60]. The content of these schemes varies by prefecture. For example, Nagano Prefecture provides subsidies (between 300 thousand and one million JPY) for households that build houses that are beneficial for the environment and human health. The policy in Gifu Prefecture is to lower the housing loan rate for households that build environment-friendly (energy-saving) houses. The housing loan rate is reduced by one percentage point for five years for households that use housing loans from private financial institutions to build houses with high-energy-saving performance.

### 2.2. Panel Data Analysis

In this study, we analyzed the causal effect of policy measures on promoting the construction of LLQH by using regression models. The general form of the models is shown in Equation (1).

(1) $$lqh_{it}=\underset{p}{\sum }\beta_{1p}policy_{pit}+\underset{c}{\sum }\beta_{2c}X_{cit}+\alpha +\theta_{i}+\zeta_{t}+\epsilon_{it}$$

where *lqh_it_* is the rate of newly built LLQH per total newly built houses in prefecture *i* in year *t.* This study considers only independent housing and not apartments. *policy_pit_* are the policy measures *p* intended to promote the building of LLQH, *X_cit_* are the control variables *c*, *α* is a constant, *θ_i_* is the prefecture-fixed effects, *ζ_t_* is the year-fixed effects, *ε_it_* is an error term, and *β*_1*p*_ and *β*_2*c*_ are the coefficients. The fixed effects *θ_i_* and *ζ_t_* are allowed to be correlated with other variables. Prefecture-fixed effects capture unobserved regional characteristics through time, including historical preferences for housing types. Year-fixed effects capture the unobserved factors that affect housing types equally across prefectures but differently by year, such as changes in the national demand for LLQH in Japan.

We conducted three types of analysis using the models based on Equation (1) with different independent variables for *policy*. First, to understand the pure effect of the policy measures promoting the construction of LLQH, we considered the policy measures that only cover LLQH (Case 1). In this case, *d_ss*1 (a dummy variable for policy measures that only cover LLQH at the prefectural level, which takes a value of one when measures exist and zero otherwise; because the types of prefectural measures vary, we used dummy variables *d_ss*1 and *d_ss*2), *l_lqh* (subsidies for LLQH in ten thousand JPY), and *l_itd* (income tax deduction for a housing loan in ten thousand JPY) were used. In addition, both *l_lqh* and *l_itd* directly provide monetary incentives to households building LLQH, so we also conducted an analysis by aggregating the two variables as *l_all* (in ten thousand JPY). 

However, the policy measures that only cover LLQH are limited, particularly at the prefectural level. Therefore, in the second analysis, we also applied policy measures that cover a broader category of housing, such as LLQH and highly insulated houses, as *policy* variables (Case 2). These variables are *d_ss*2 (a dummy variable for policy measures at the prefectural level that takes a value of one when the measures exist and zero otherwise), *l_lqh*, *l_itd*, *s_shs* (Subsidies for Housing Stock Circulation Support in ten thousand JPY), *s_hcb* (Housing Cash Benefit in ten thousand JPY), and *d_s_hep* (a dummy variable for Housing Eco-Points that takes a value of one when the measures exist and zero otherwise). In addition, similar to the first analysis, we also conducted an analysis by aggregating similar variables (i.e., aggregating *s_shs* and *s_hcb* to create *s_all* and aggregating *l_lqh* and *l_itd* to create *l_all*). 

Finally, the third analysis aggregated all the *policy* variables expressed in a monetary unit (i.e., aggregating *l_lqh*, *l_itd*, *s_shs*, and *s_hcb* to create *t_all*) (Case 3). 

Other policy measures are also used to promote the construction of LLQH, such as the deduction of registration and license taxes, real estate acquisition taxes, and fixed assets taxes (see Table 1). However, because the values for these policy measures were identical across prefectures during the study period, these variables were not applicable to this study. In the above policy variables, *d_ss*1 and *d_ss*2 are related to prefectural measures, whereas the others are related to national measures. To summarize, *d_ss*1, *l_lqh*, *l_itd* and *l_all* were used for Case 1; *d_ss*2, *l_lqh*, *l_itd*, *l_all*, *s_shs*, *s_hcb*, *s_all* and *d_s_hep* were used for Case 2; and *d_ss*2, *d_s_hep*,and *t_all* were used for Case 3 for *policy*.

As the control variables for all analyses, we used the number of households (*hld* in thousand household), household income in year *t* (*hic* in ten thousand JPY), accumulated amount of household savings (*sav* in ten thousand JPY), land price (*ldp* in thousand JPY/m^2^), and housing loan rate (*hlr* in %). The number of households is used to remove the effect of the scale of households on the dependent variable. Household income and savings are considered to affect households’ choice of housing because these variables are related to household wealth. Land price and housing loan rate, which directly link to households’ expenditure on housing, are also considered to affect households’ choice of housing. In this study, we focused on the demand side for the selection of the independent variables because (1) we concentrated on households’ choice, and (2) data for the supply side (e.g., the costs for building LLQH) were not available by year and prefecture.

Table 3 summarizes the variables used in this study and their explanations.

For the dependent variable, we used the rate of newly built LLQH instead of the number of newly built LLQH, as shown in Equation (1). If we use the latter, the result is affected by the total number of newly built houses (the denominator of *lqh*). Therefore, to remove this influence and confirm the impact of the independent variables, particularly the policy variables, we used the rate rather than the number.

In this study, we used panel data for 47 prefectures across seven years from 2010 to 2016. The sample size was 329. Although the policy measures were introduced in 2009, they began in the middle of the year. Therefore, we did not use data for 2009. We utilized Stata 15.1 software to conduct the analyses.

### 2.3. Data

The data used in the regression models include the variables explained in Section 2.2. The unit of observation is the prefecture; 47 prefectures exist in Japan. The dataset used in this study is a balanced panel of 329 samples consisting of 47 prefectures for seven years (annual data); thus, there is no attrition. The descriptive statistics and data sources are shown in Table 4. As explained in Section 2.2, *d_ss*1, *d_ss*2, and *d_s_hep* are dummy variables. The data in monetary units are provided in real terms using deflators reported by the Cabinet Office of the Government of Japan [61].

**Figure 1:**
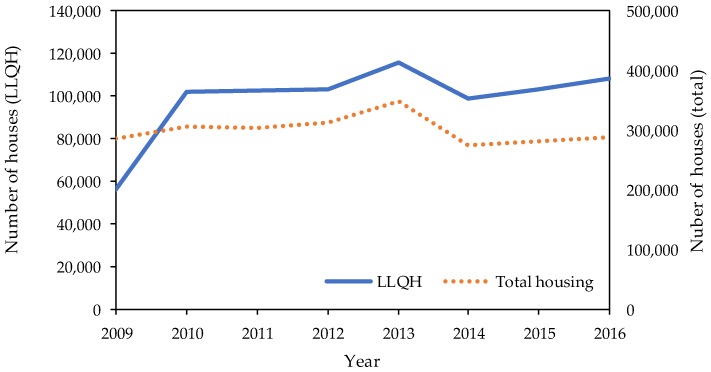
Number of houses (long-life quality housing (LLQH) and total housing) newly built in each year.

**Figure 2:**
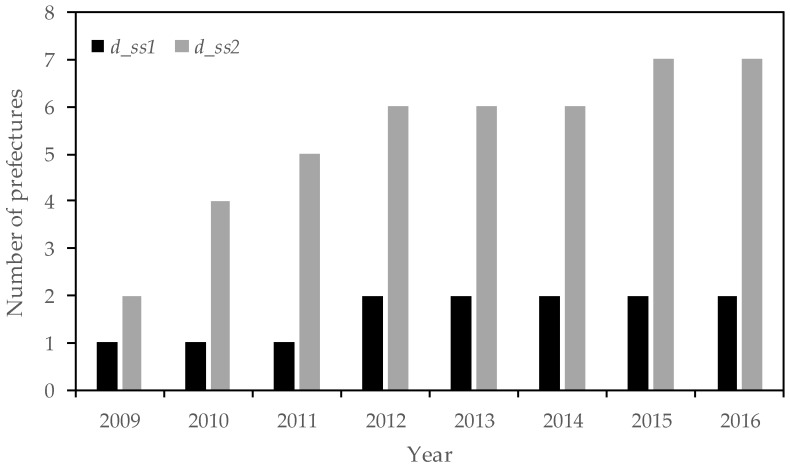
Number of prefectures introducing their own policy measures for LLQH. *d_ss*1 shows prefectures introducing policy measures that only cover LLQH, whereas *d_ss*2 shows prefectures introducing policy measures that cover LLQH and other types of housing.

Figure 1 shows the trend of the number of newly built houses (LLQH and total) for each year. Both categories show similar trends. The number of newly built LLQH was less than 60,000 in 2009, which was the year the scheme started (not used in the analyses). However, the number increased to around 100,000 to 110,000 in 2010. Figure 2 describes the changes in policy implementation in prefectures between 2009 and 2016.

## 3. Results and Discussion

Table 5 shows the results for Case 1. All standard errors are clustered at the prefectural level. The main results of our analyses were fixed-effect (FE) models, but we also showed the results of pooled ordinary least squares (OLS) (column 1) and the analysis without the control variables (column 2). Columns 3–6 show the FE models. Observation of the policy-related variables shows that income tax deduction for a housing loan (*l_itd*) or an aggregation of *l_itd* and *l_lqh* (*l_all*) was positive and statistically significant. However, although subsidies for LLQH (*l_lqh*) were statistically significant in column 2, this was not true in columns 3 and 4, in which the prefecture FE or the prefecture and year FE were considered, although the coefficients were positive. Prefectural measures (*d_ss*1) were not statistically significant for all analyses when including the control variables and FE. These results suggest that the national-level measures were mostly effective in increasing the ratio of newly built LLQH, whereas the prefectural-level measures were not. One reason why the prefectural measures were not statistically significant might be the very limited number of prefectures that introduced measures (Figure 2). As explained in Section 2.1, policy measures corresponding to *d_ss*1 were introduced in Fukuoka and Shizuoka Prefectures. Thus, the effect of prefectural measures (*d_ss*1) was not precisely determined. In addition, compared with the national measures, fewer households might have been aware of the prefectural measures, which could have affected the results.

Table 6 shows the results for Case 2. For Cases 2 and 3, we only show the models with FE and explain the results of the models with double FE. The procedure for the analyses for Case 2 was the same as that for Case 1, but additional *policy* variables (i.e., variables related to LLQH but not limited to them) were introduced in Case 2. The results of Case 2 were similar to those of Case 1. Within the policy measures that only cover LLQH (i.e., the same variables as in Case 1), the subsidy for LLQH (*l_lqh*) was positive but not statistically significant, whereas the income tax deduction for a housing loan (*l_itd*) was statistically significant in column 8. As a result, the aggregated term *l_all* was also statistically significant for the analysis shown in column 10. These results were consistent with those in Case 1.

Within the policy measures that were related to LLQH but not limited to them, all the variables (Subsidies for Housing Stock Circulation Support (*s_shs*) and Housing Cash Benefit (*s_hcb*)) were not statistically significant if both prefecture and year FE were considered (column 8). Furthermore, the aggregated term *s_all* was not statistically significant, as shown in column 10. The prefectural measures (*d_ss*2) were also not statistically significant, as in Case 1.

The results from Cases 1 and 2 suggest that the former type of policy measure (i.e., those only related to LLQH) was more influential in increasing the ratio of LLQH than was the latter type. This may be because the former measures focused on subsidizing LLQH, meaning that they are simpler to understand for households than the latter and are utilized by households building LLQH. In addition, the results from the double FE estimations in Cases 1 and 2 indicate that tax deductions strongly affected the ratio of LLQH. These findings may reflect the fact that the benefit households receive is larger for tax deductions than for subsidies. Because of this, the effectiveness of the policy measures was different by type.

Finally, Table 7 shows the results for Case 3. Similar to Case 2, these analyses also include both types of policy measures. However, all similar measures at the national level (measures expressed in monetary units) were aggregated into one variable (*t_all*, aggregation of *l_lqh*, *l_itd*, *s_shs*, and *s_hcb*). The aggregated variable *t_all* was positive and statistically significant, whereas the two other variables (prefectural measures [*d_ss*2] and Housing Eco-Points [*d_s_hep*]) were not.

From these three cases, it was determined that policy measures (economic incentives) introduced at the prefectural level were not effective in increasing the ratio of LLQH. However, the effect of the national-level measures differed by measure. Overall, the national-level measures increased the ratio of LLQH, as shown in Case 3. However, observation of the measures individually shows that the effect was different. The measures that covered only LLQH, particularly tax deductions, tended to have more significant effects than those covering both LLQH and other types of housing (Case 2). However, it should be noted that the coefficients were not so large, as shown in Table 5, Table 6 and Table 7. Such measures were therefore basically effective. There are potential biases because of the introduction of policy measures, such as consumers’ moving to another prefecture where policies are implemented, although prefecture and year dummies could remove prefecture and year fixed effects. However, tax incentives are not the reason of migration in Japan [69], so such biases did not occur in this case.

Observation of the control variables shows that overall, the results were similar in all cases. The coefficients for the number of households (*hld*) were positive and statistically significant. This can be interpreted that as the number of households increases, more households choose to build LLQH. For the variables related to household wealth, the coefficients for household income (*hic*) were not statistically significant, whereas the coefficients for household saving (*sav*) were negative and statistically significant. This finding means that the monetary flow did not affect household decisions to choose LLQH when building new houses. Conversely, the results indicate that households with more savings tended to choose building regular houses. This might be due to people’s expectation that LLQH involves reduced utility costs, such as heating expenses. Thus, households with less savings would possibly prefer LLQH over regular housing. The coefficients for land price (*ldp*) were consistently negative but not statistically significant. Although land price was expected to have a negative impact because building LLQH, which is costlier than regular houses, became more expensive on more expensive land, meaning that households tended to choose to build regular houses, the results did not show such consequences. Finally, the coefficients for the housing loan rate (*hlr*) were also not statistically significant. Although the housing loan rate was expected to have a negative impact because building LLQH, which is costlier than regular houses, became more expensive on a higher loan rate, meaning that households tended to choose building regular houses, the results did not show such consequences.

## 4. Conclusions

Using panel data from 47 prefectures across seven years, we analyzed the factors affecting Japanese households’ decisions to build LLQH. We found that policy measures covering only LLQH, particularly tax deductions, were generally effective in promoting the construction of LLQH, and such policy measures were more effective than those covering both LLQH and other types of housing. National-level measures tended to be effective, whereas prefectural-level measures were not. In addition, although the effects of individual measures differed, the overall effect of policy measures was confirmed. From these results, it can be concluded that providing economic incentives has had positive impacts on promoting LLQH, although the effect is not very large. Because not all policy measures were effective in promoting LLQH, it was considered that more LLQH would have been constructed had monetary resources been concentrated on the measures that had significant effects.

As a step toward a sustainable and healthy society, it would be prudent to continue providing economic incentives to households that build LLQH in order to further increase LLQH, which comprised only around 14% of newly built houses between 2010 and 2016 (see Table 4). However, such policy measures have limitations. Not all the measures were effective, and continuously providing subsidies or deductions is also not possible. On the point that not all the measures were effective, concentrating monetary resources on measures that are more effective in promoting LLQH would be reasonable. For example, policy measures that cover only LLQH were more effective than those covering both LLQH and other housing types at the national level. Thus, focusing monetary resources on measures covering only LLQH would be more efficient. For the latter, subsidies or tax deductions cannot be continued in the long term. Because such economic incentives are meant to reduce the cost of building LLQH, making efforts to reduce the costs of LLQH instead of providing economic incentives via policy is essential.

Increasing the number and share of LLQH or similar types of housing, such as sustainable housing, is essential for building a sustainable and healthy society in the residential sector on a global scale. The above-mentioned implications (i.e., providing economic incentives) may also be applicable to other countries with regard to increasing the amount and share of sustainable housing. In particular, subsidies/tax deductions are important for promoting this type of housing in countries with short average house lifetimes (similar to Japan), such as Korea [21]. However, because characteristics (e.g., response to economic incentives), situations (e.g., economic situations), and policy systems (e.g., subsidies and taxes) differ by country, similar analyses are needed for other countries to further understand and confirm the factors that promote sustainable housing.

Finally, several limitations of this study should be mentioned. First, we focused only on demand-side analysis. Although we ran several regression models and found the effect of policy implementation on the LLQH market, the LLQH market could also depend on the supply side because the supplier would be able to promote LLQH to its customers. However, because supply-side data were not available, this study could not attempt analyses with supply-side variables. The second limitation is related to the variables for policy measures. For the prefectural-level policy measures, we applied dummy variables because various types of measure have been introduced in each prefecture. However, analyzing these measures more precisely is necessary to further understand the effect of prefectural-level policy measures. Future studies should address these issues.

## Figures and Tables

**Table 1:** Preferential measures for taxes for LLQH.

Type of Tax		Regular Housing	LLQH
Registration and license tax	Ownership preservation registration	0.15%	0.1%
	Ownership transfer registration	0.3%	0.2%
Real estate acquisition tax		12 million JPY deduction	13 million JPY deduction
Fixed assets tax		Half of full tax rate (3 years)	Half of full tax rate (5 years)

**Table 2:** Preferential measures for housing loans for LLQH.

		2009	2010	2011	2012	2013	2014–
Regular housing	Deduction rate	1.0%					
	Maximum annual deduction (thousand JPY)	500	500	400	300	200	400
LLQH	Deduction rate	1.2% (2009–2011)			1.0% (2012–2014)		
	Maximum annual deduction (thousand JPY)	600	600	600	400	300	500

Notes: The years show the year that residents started living in a house, and the deduction continues for 10 years. The corresponding deduction rate is applied for newly built housing, but the maximum deduction amount is also determined.

**Table 3:** Summary of variables and their explanations.

Variables	Explanation
*lqh*	The rate of newly built LLQH per total newly built houses
*d_ss*1	A dummy variable for policy measures that only cover LLQH at the prefectural level
*d_ss*2	A dummy variable for policy measures that cover LLQH and other types of housing at the prefectural level
*l_lqh*	Subsidies for LLQH (ten thousand JPY)
*l_itd*	Income tax deduction for a housing loan (ten thousand JPY)
*l_all*	Aggregation of *l_lqh* and *l_itd* (ten thousand JPY)
*s_shs*	Subsidies for Housing Stock Circulation Support (ten thousand JPY)
*s_hcb*	Housing Cash Benefit (ten thousand JPY)
*s_all*	Aggregation of *s_shs* and *s_hcb* (ten thousand JPY)
*d_s_hep*	A dummy variable for Housing Eco-Points
*t_all*	Aggregation of *l_lqh*, *l_itd*, *s_shs*, and *s_hcb* (ten thousand JPY)
*hld*	Number of households (thousand household)
*hic*	Household income in year *t* (ten thousand JPY)
*sav*	Accumulated amount of household savings (ten thousand JPY)
*ldp*	Land price (thousand JPY/m^2^)
*hlr*	Housing loan rate (%)

**Table 4:** Descriptive statistics and data sources of the variables.

Variables	Mean	Std. Dev.	Min	Max	Data Sources
*lqh*	0.306	0.114	0.0340	0.693	[62,63]
**Policy Measures (*policy*)**					
*d_ss*1	0.036	0.188	0	1	a
*d_ss*2	0.125	0.331	0	1	a
*l_lqh*	114.307	17.739	101.967	150.873	a
*l_itd*	498.486	99.268	314.560	622.200	[64]
*l_all*	612.793	100.676	419.414	725.900	b
*s_shs*	7.184	17.625	0	50.291	[50]
*s_hcb*	13.052	15.095	0	30.924	[51]
*s_all*	20.236	28.023	0	80.466	b
*d_s_hep*	0.714	0.452	0	1	[52]
*t_all*	633.029	110.062	419.414	734.248	b
**Control Variables (*X*)**					
*hld*	1173.892	1257.816	226.434	6889.913	[65]
*hic*	625.358	57.062	463.867	774.946	[66]
*sav*	1669.096	401.594	599.386	3015.447	[66]
*ldp*	52.205	53.082	13.724	334.737	[67]
*hlr*	2.290	0.540	1.371	2.938	[68]

Note: a: Survey by websites of prefectures and inquiry on prefectures; b: our own calculation.

**Table 5:** Results of the panel data analyses for Case 1.

Items	(1)	(2)	(3)	(4)	(5)	(6)
**Policy Measures (*policy*)**						
*d_ss*1	0.0156	0.0198 ***	0.00335	0.00323	0.00329	0.00323
	(0.0191)	(0.00388)	(0.00510)	(0.00521)	(0.00515)	(0.00521)
*l_lqh*	8.19 × 10^−5^	0.000499 ***	0.000120	1.59 × 10^−5^	-	-
	(0.000147)	(0.000119)	(9.59 × 10^−5^)	(0.000129)		
*l_itd*	9.63 × 10^−5^ ***	6.47 × 10^−5^ ***	0.000102 ***	0.000113 ***	-	-
	(1.49 × 10^−5^)	(1.40 × 10^−5^)	(8.54 × 10^−6^)	(1.93 × 10^−5^)		
*l_all*	-	-	-	-	0.000102 ***	0.000108 ***
					(9.33 × 10^−6^)	(1.43 × 10^−5^)
**Control Variables (*X*)**						
*hld*	1.07 × 10^−7^ ***	-	3.06 × 10^−7^ ***	3.06 × 10^−7^ ***	3.06 × 10^−7^ ***	3.06 × 10^−7^ ***
	(1.81 × 10^−8^)		(6.14 × 10^−8^)	(6.62 × 10^−8^)	(6.12 × 10^−8^)	(6.62 × 10^−8^)
*hic*	4.33 × 10^−5^	-	5.14 × 10^−5^	5.59 × 10^−5^	5.15 × 10^−5^	5.59 × 10^−5^
	(4.63 × 10^−5^)		(3.64 × 10^−5^)	(3.75 × 10^−5^)	(3.64 × 10^−5^)	(3.75 × 10^−5^)
*sav*	−8.94 × 10^−6^	-	−1.97 × 10^−5^ ***	−1.99 × 10^−5^ ***	−1.97 × 10^−5^ ***	−1.99 × 10^−5^ ***
	(7.54 × 10^−6^)		(6.31 × 10^−6^)	(6.47 × 10^−6^)	(6.31 × 10^−6^)	(6.47 × 10^−6^)
*ldp*	−1.34 × 10^−6^ ***	-	−1.62 × 10^−6^ *	−1.57 × 10^−6^	−1.61 × 10^−6^ *	−1.57 × 10^−6^
	(4.26 × 10^−7^)		(8.59 × 10^−7^)	(1.04 × 10^−6^)	(8.44 × 10^−7^)	(1.04 × 10^−6^)
*hlr*	−0.00873	-	0.00184	[omitted]	0.00128	0.00320
	(0.00556)		(0.00573)		(0.00453)	(0.00477)
Constant	0.200 ***	0.216 ***	−0.0369	−0.0295	−0.0344	−0.0455
	(0.0409)	(0.0101)	(0.0824)	(0.0754)	(0.0798)	(0.0815)
Estimation	Pooled OLS	FE	FE	FE	FE	FE
Single FE		X	X		X	
Double FE				X		X
Observations	329	329	329	329	329	329
Adjusted R^2^	0.363	0.191	0.451	0.455	0.450	0.455

Notes: Heteroskedasticity-robust standard errors are given in parentheses for the pooled OLS model in column 1. Standard errors clustered at the prefectural level are shown in parentheses for FE estimations. *** *p* < 0.01, ** *p* < 0.05, * *p* < 0.1. Single FE models include the FE of prefectures, whereas double FE models include the FE of prefectures and the years. The dependent variable in all models is *lqh*. One variable was omitted (with “[omitted]” in the table) during the analysis because of collinearity (i.e., the variable was constant within groups).

**Table 6:** Results of the panel data analyses for Case 2.

Items	(7)	(8)	(9)	(10)
**Policy Measures (*policy*)**				
*d_ss*2	−0.00700	−0.00700	−0.00792	−0.00700
	(0.0148)	(0.0148)	(0.0145)	(0.0148)
*l_lqh*	−0.00315	0.000179	-	-
	(0.00247)	(0.000118)		
*l_itd*	8.86 × 10^−5^	0.000113 ***	-	-
	(0.000129)	(2.03 × 10^−5^)		
*l_all*	-	-	0.000103 ***	0.000112 ***
			(1.38 × 10^−5^)	(2.08 × 10^−5^)
*s_shs*	0.00118	−8.29 × 10^−5^	-	-
	(0.000763)	(8.13 × 10^−5^)		
*s_hcb*	−0.000199	−0.000106	-	-
	(0.00190)	(0.000170)		
*s_all*	-	-	−4.51 × 10^−5^	−5.19 × 10^−5^
			(0.000146)	(8.85 × 10^−5^)
*d_s_hep*	−0.0922	[omitted]	−0.00222	[omitted]
	(0.0626)		(0.00330)	
**Control Variables (*X*)**				
*hld*	3.05 × 10^−7^ ***	3.05 × 10^−7^ ***	3.04 × 10^−7^ ***	3.05 × 10^−7^ ***
	(6.58 × 10^−8^)	(6.58 × 10^−8^)	(6.27 × 10^−8^)	(6.58 × 10^−8^)
*hic*	5.56 × 10^−5^	5.56 × 10^−5^	5.25 × 10^−5^	5.56 × 10^−5^
	(3.74 × 10^−5^)	(3.74 × 10^−5^)	(3.66 × 10^−5^)	(3.74 × 10^−5^)
*sav*	−1.98 × 10^−5^ ***	−1.98 × 10^−5^ ***	−1.95 × 10^−5^ ***	−1.98 × 10^−5^ ***
	(6.43 × 10^−6^)	(6.43 × 10^−6^)	(6.28 × 10^−6^)	(6.43 × 10^−6^)
*ldp*	−1.57 × 10^−6^	−1.57 × 10^−6^	−1.55 × 10^−6^	−1.57 × 10^−6^
	(1.04 × 10^−6^)	(1.04 × 10^−6^)	(9.27 × 10^−7^)	(1.04 × 10^−6^)
*hlr*	−0.00452	[omitted]	1.45 × 10^−5^	[omitted]
	(0.0740)		(0.0116)	
Constant	0.415	−0.0444	−0.0290	−0.0372
	(0.421)	(0.0783)	(0.0819)	(0.0781)
Estimation	FE	FE	FE	FE
Single FE	X		X	
Double FE		X		X
Observations	329	329	329	329
Adjusted R^2^	0.456	0.456	0.452	0.456

Notes: Standard errors clustered at the prefectural level are shown in parentheses. *** *p* < 0.01, ** *p* < 0.05, * *p* < 0.1. Single FE models include the FE of prefectures, whereas double FE models include the FE of prefectures and the years. The dependent variable in all models is *lqh*. Some variables were omitted (with “[omitted]” in the table) during the analysis because of collinearity (i.e., these variables were constant within groups).

**Table 7:** Results of the panel data analyses for Case 3.

Items	(11)	(12)
**Policy measures (*policy*)**		
*d_ss*2	−0.00770	−0.00700
	(0.0146)	(0.0148)
*d_s_hep*	−0.00325	[omitted]
	(0.00316)	
*t_all*	8.93 × 10^−5^ ***	9.76 × 10^−5^ ***
	(8.56 × 10^−6^)	(1.31 × 10^−5^)
**Control variables (*X*)**		
*hld*	3.06 × 10^−7^ ***	3.05 × 10^−7^ ***
	(6.09 × 10^−8^)	(6.58 × 10^−8^)
*hic*	4.98 × 10^−5^	5.56 × 10^−5^
	(3.66 × 10^−5^)	(3.74 × 10^−5^)
*sav*	−1.97 × 10^−5^ ***	−1.98 × 10^−5^ ***
	(6.30 × 10^−6^)	(6.43 × 10^−6^)
*ldp*	−1.68 × 10^−6^ *	−1.57 × 10^−6^
	(8.57 × 10^−7^)	(1.04 × 10^−6^)
*hlr*	0.00790 *	0.00824
	(0.00455)	(0.00534)
Constant	−0.0355	−0.0509
	(0.0793)	(0.0817)
Estimation	FE	FE
Single FE	X	
Double FE		X
Observations	329	329
Adjusted R^2^	0.451	0.373

Notes: Standard errors clustered at the prefectural level are shown in parentheses. *** *p* < 0.01, ** *p* < 0.05, * *p* < 0.1. Single FE models include the FE of prefectures, whereas double FE models include the FE of prefectures and the years. The dependent variable in all models is *lqh*. One variable (with “[omitted]” in the table) was omitted during the analysis because of collinearity (i.e., the variable was constant within groups).
